# Scan-Free Absorbance Spectral Imaging *A*(*x*, *y*, λ) of Single Live Algal Cells for Quantifying Absorbance of Cell Suspensions

**DOI:** 10.1371/journal.pone.0128002

**Published:** 2015-06-10

**Authors:** Takumi Isono, Kyohei Yamashita, Daisuke Momose, Hiroki Kobayashi, Masashi Kitamura, Yusuke Nishiyama, Takahiro Hosoya, Hiroaki Kanda, Ayane Kudo, Norihide Okada, Takafumi Yagi, Kazuaki Nakata, Shigeru Mineki, Eiji Tokunaga

**Affiliations:** 1 Department of Physics, Faculty of Science, Tokyo University of Science, 1-3 Kagurazaka, Shinjuku-ku, Tokyo 162-8601, Japan; 2 Department of Applied Biological Science, Faculty of Science and Technology, Tokyo University of Science, 2641 Yamazaki, Noda-shi, Chiba-ken 278-8510, Japan; 3 Research Center for Green and Safety Sciences, Tokyo University of Science, 1-3 Kagurazaka, Shinjuku-ku, Tokyo 162-8601, Japan; Texas A&M University, UNITED STATES

## Abstract

Label-free, non-invasive, rapid absorbance spectral imaging *A*(*x*,*y*,λ) microscopy of single live cells at 1.2 *μ*m × 1.2 *μ*m resolution with an NA = 0.85 objective was developed and applied to unicellular green algae *Chlamydomonas reinhardtii*. By introducing the fiber assembly to rearrange a two-dimensional image to the one-dimensional array to fit the slit of an imaging spectrograph equipped with a CCD detector, scan-free acquisition of three-dimensional information of *A*(*x*,*y*,λ) was realized. The space-resolved absorbance spectra of the eyespot, an orange organelle about 1 *μ*m, were extracted from the green-color background in a chlorophyll-rich single live cell absorbance image. Characteristic absorbance change in the cell suspension after hydrogen photoproduction in *C. reinhardtii* was investigated to find a single 715-nm absorption peak was locally distributed within single cells. The formula to calculate the absorbance of cell suspensions from that of single cells was presented to obtain a quantitative, parameter-free agreement with the experiment. It is quantitatively shown that the average number of chlorophylls per cell is significantly underestimated when it is evaluated from the absorbance of the cell suspensions due to the package effect.

## Introduction

Microalgae, photosynthetic unicellular organisms, are collecting global attention from their high potentials for resources of biofuel and food [1–4]. Precise knowledge of absorptive properties of them to sunlight is vitally important for seeking for efficient photoproduction of renewable energy from microalgae [5, 6]. It is well known that a suspension of absorbing cells which contain densely packed pigments exhibit a flattened absorbance spectrum compared with that of a solution containing the same average number density of pigments as homogeneous dispersion;the higher the absorption of the individual cells, the stronger the flattening. This nonlinearity results in the ‘package’ effect [7, 8], which also can be seen as a reduction in the absorption of pigmented cells relative to the absorption of the same pigments in solution [9]. However, there has been no fully quantitative evaluation of absorbance of cell suspensions on the basis of absorbance of single cells. Detailed theoretical modeling of light attenuation properties including scattering effects by phytoplanktonic cells was also previously presented [10], but single-cell absorbance is usually left for an unknown fitting parameter because of lack of a knowledge on detailed absorptive properties of single live cells.

For early 1960’s, there was a pioneering work on absorption spectroscopy of a single live cell [11], but afterward advances in dynamic live-cell imaging based on fluorescence confocal microscopy are so impressive and successful in medical and biological science while absorption imaging is not fully explored except for a few examples such as one of variations of hyperspectral approach [12, 13].

In this paper, we introduce a live-cell imaging method using absorption microspectroscopy. In addition to characterization of absorptive properties of cells, there are several reasons which necessitate absorbance spectral imaging of live algal cells: Firstly, plant cells have cell walls which make it difficult to introduce fluorescent labels into the cells. Secondly, existence of photosynthetic pigments, chlorophyll, which fluoresce red, prevent the use of red fluorescent labels. Third, what is most important, absorption spectra contain much more information than fluorescence spectra about the excited states of cellular organisms and pigments, because the latter usually give only the information of the lowest (relaxed) excited state. Fourth, there are no fluorescent labels (fluorophores) needed, which may affect biochemical properties of the cells, to realize a noninvasive measurement in the true sence of the word.

Fluorescence imaging has, of course, fundamental advantages over absorbance imaging in that fluorescence from single molecules is detectable while absorbance of single molecules is hard to be detected because the former is background-free measurement while the latter suffers from noise of background light. Therefore variations based on single-molecule fluorescence such as PALM(photoactivated localization microscopy) [14] are advantages of fluorescence imaging.

## Materials and Methods

The sample used was the green alga *Chlamydomonas* *reinhardtii* Dangeard NIES-2238 (IAM C-541), which is one of the model photosynthetic micro-organisms [15] and also attractive in view of its ability of hydrogen photoproduction [16, 17]. The absorbance spectra of cell suspensions were measured with an absorption spectrophotometer using an integrating sphere (SolidSpec-3700DUV, Shimadzu). All the measurements were performed at room temperature.

### Absorption Spectral Imaging A(x,y,*λ*)

**Fig 1:**
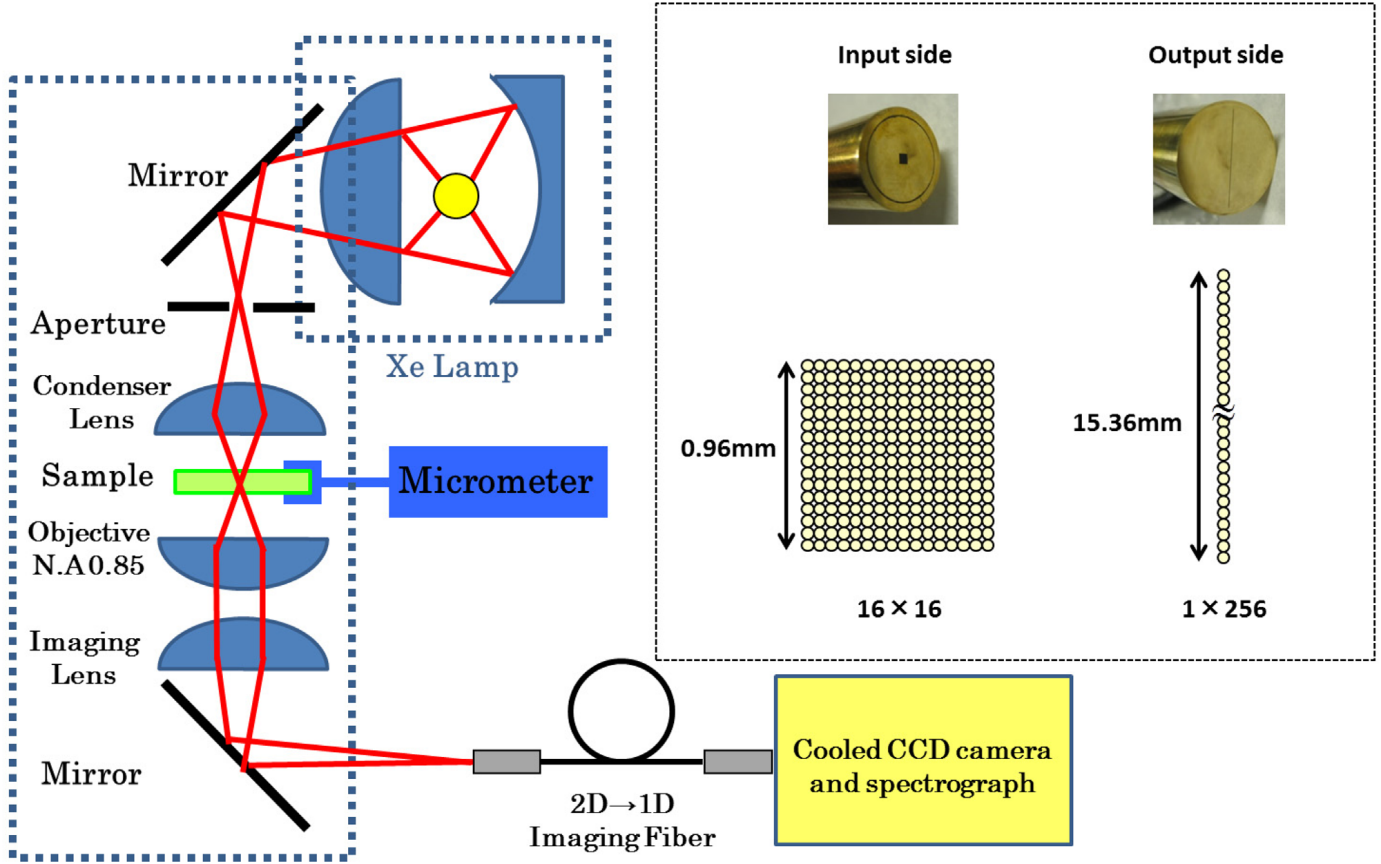
Experimental setup for absorbance spectral imaging of single cells.

Experimental setup is shown in Fig 1. Since *Chlamydomonas* has two flagella, its mobility should be suppressed for live-cell imaging. For this purpose, a culture solution suspending cells was mixed with glycerin to be set on a hollow slide glass, or agar was added to the solution to exert fixing pressure upon cells and the solution was set on a hemacytometer plate or on a slide glass. For both cases, the solution was covered with a cover glass. The samples were set on an inverted microscope (IX71, OLYMPUS) and observed with a ×100 objective lens of NA0.85 from below. The light source was a 75 W xenon lamp (Ushio) or a 150 W Xenon lamp (Hamamatsu) to illuminate the sample from above through a condenser. The intensity on the sample was ca. 1.3 W/cm^2^ and 650 nW per cell for 1 second exposure and 0.040 W/cm^2^ 20 nW/cell for 10 second exposure (assuming 8 *μ*m diameter). The transmitted light was transferred through the objective and a focusing lens to the side port of the microscope.

#### Scanning Slit(Line) Image Method

On the side port of the microscope, the ×100 magnified image of the sample was focused on the entrance slit of an *f* = 35 cm or *f* = 10 cm imaging spectrometer (MS3504i, SOLAR TII). The slit image of the zeroth-order diffraction was observed with an electrically-cooled EM-CCD camera (1600 × 400 pixels with 16 *μ*m pixel size, DU971N-UVB, Newton, Andor) to identify the full image of a single cell. A vertical region (*y*-axis) with 1.0 *μ*m horizontal width which includes a partial image of the single cell was selected by narrowing the slit width to 100 *μ*m and it is dispersed spectrally (*λ*) by rotating the grating of 300gr/500nm to the first-order diffraction angle. The measurement time for the transmitted light spectrum was ca. 1 seconds. This process is repeated by shifting the cell image horizontally (*x*-axis) on the slit using a high precision micrometer stage by 1 *μ*m step to cover the full image of the cell. The absorbance spectrum was calculated from the spectrum thus obtained using the spectrum of the light transmitted through a region outside the cell.

#### 2D-image to 1D(Slit)-image Conversion Method

It takes time to scan the slit image horizontally for imaging the whole cell. For a 10 *μ*m cell, for example, at least 10-times scan is needed, during which the living cell might move from its original position and pigments might be photodamaged due to long irradiation time of the white light. To overcome these disadvantages, we introduced a fiber bundled array to convert a 2-dimensional image to the 1-dimensional slit image as shown in Fig 1. A 50-*μ*m core/55-*μ*m clad/60-*μ*m coating silica fibers are assembled to 16×16 (0.96×0.96 mm^2^) array on the input side and to 1×256 (0.06×15.36 mm^2^) array on the output (slit) side. On the side port of the microscope, the ×100 magnified image of the sample was focused on the 16×16 2D array (*x* × *y*), which is rearranged in order into the 1D array to fit the entrance slit of the spectrometer (MS3504i or SL100M, SOLAR TII). Thus, in principle, 256 spectra can be simultaneously obtained on the EM-CCD camera through the spectrometer for a single exposure time. In fact, however, the vertical CCD size was only 6.4 mm such that three-times (SL100M) or four-times (MS3504i, where the slit image is 1.5 times magnified on the CCD) measurement is needed by shifting the 1D-array on the slit vertically. Therefore the compression of the total exposure time was ca. one-third of that for the scanning slit method. This could be improved if a CCD with a longer vertical size and the imaging spectrometer with a longer vertical imaging area more than 16 mm. In this method, theoretical spatial resolution of optics is 0.61× 550 (nm)/0.85 ≈ 400 nm, so that the experimental resolution should be larger than 600 nm, the value limited by the fiber size of 60 *μ*m. The resolution was experimentally estimated to be about 1.2 *μ*m from the absorbance image of an eyespot [Appendix 1].

### Evaluation of Cell Number density and Cell Diameter

In Sec. IV, we evaluate the absorbance of a cell suspension from that of single cells. For this procedure to be reliable, the cell number density *n*_c_ and the chloroplast average diameter *d*_ave_(= *d*) in the cell suspension need to be evaluated accurately. We initially used a commercially available hemacytometer to count cells and to measure the diameters of them by the eye, but we found there are significant fluctuations in both values owing to the insufficient sample quantity (an order of 1000 cells for *n*_c_ and an order of 10 cells for *d*). Therefore we developed the original digital photo analysis program to count the number of the sampled cells and to evaluate the average diameter of them to increase the sample quantity (orders of 100000 and 1000 cells for *n*_c_ and *d*, respectively). A 10 *μ*L of the cell suspension was dropped on a slideglass with a micropipette to be spread out and covered with a cover slip of 18 mm×18 mm for observation with a microscope. Randomly sampled 599 images of 1.1 mm×0.8 mm area on 10 präparats were selected and the number of cells per area was counted by the program. The total count reached 211663 cells to obtain 353.36 cells as the average number per image. As a result, we obtained the average number density of cells in the suspension as *n*_c_ = (1.67 ± 0.04) × 10^4^/mm^3^. The standard deviation was significantly (by 10 times) larger than that expected for a Poisson distribution, $0.004\times 10^{4}/{\text{mm}}^{3}\left(=16700\times \sqrt{211663}/211663\right)$. In order to assure 3% precision in the cell number density estimation, therefore, the counted number is needed to be more than 100000. The average diameter of cells (chloroplast) was evaluated from the average of randomly sampled 846 cells as *d*_ave_ = 7.42 ± 1.53 *μ*m. The details of the experimental and analysis methods for these estimations are described in Supporting Information (S1 Text, S1–S4 Figs).

## Results and Discussion

**Fig 2:**
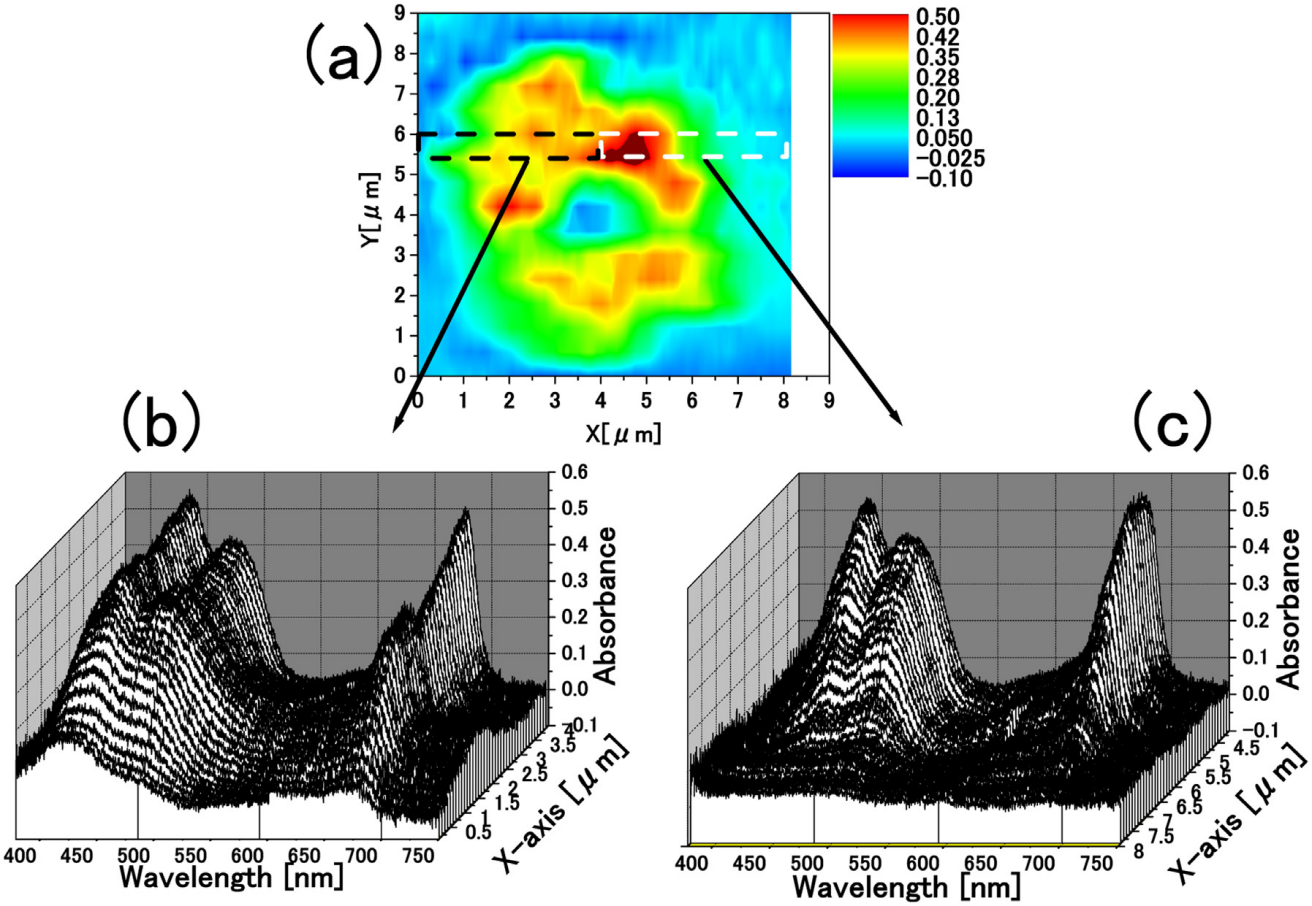
A(x,y,*λ*) obtained by the 2D-1D conversion method. (a) Absorbance image of a single living cell at *λ* = 680 nm: *A*(*x*, *y*, *λ* = 680 nm). (b) Absorbance spectra of the cell at y = 5.7 *μ*m from *x* = 0 to *x* = 4.0 *μ*m: *A*(*x*, *y* = 5.7 *μ*m, *λ*), integrated over the area of Δ*x* × Δ*y* = 0.10 *μ*m × 0.60 *μ*m for each spectrum (0.10 *μ*m × 0.60 *μ*m does not indicate spatial resolution but a division unit). (c) Absorbance spectra of the cell at y = 5.7 *μ*m from *x* = 4.0 to *x* = 8.0 *μ*m: *A*(*x*, *y* = 5.7 *μ*m, *λ*).

A spatially resolved absorbance image of a single living cell is shown in Fig 2(a) at a selected wavelength, *λ* = 680 nm, of the absorbance peak of chlorophyll *a* (Chl *a*) Q-band. One can see that Chl *a* is nonuniformly distributed within the cell [15]. A similar 2-dimensional plot of the absorbance distribution at any wavelength from 375 nm to 825 nm was obtained. Figs 2(b) and 2(c) show the position dependent spectra of 0.10 *μ*m × 0.6 *μ*m area.

**Fig 3:**
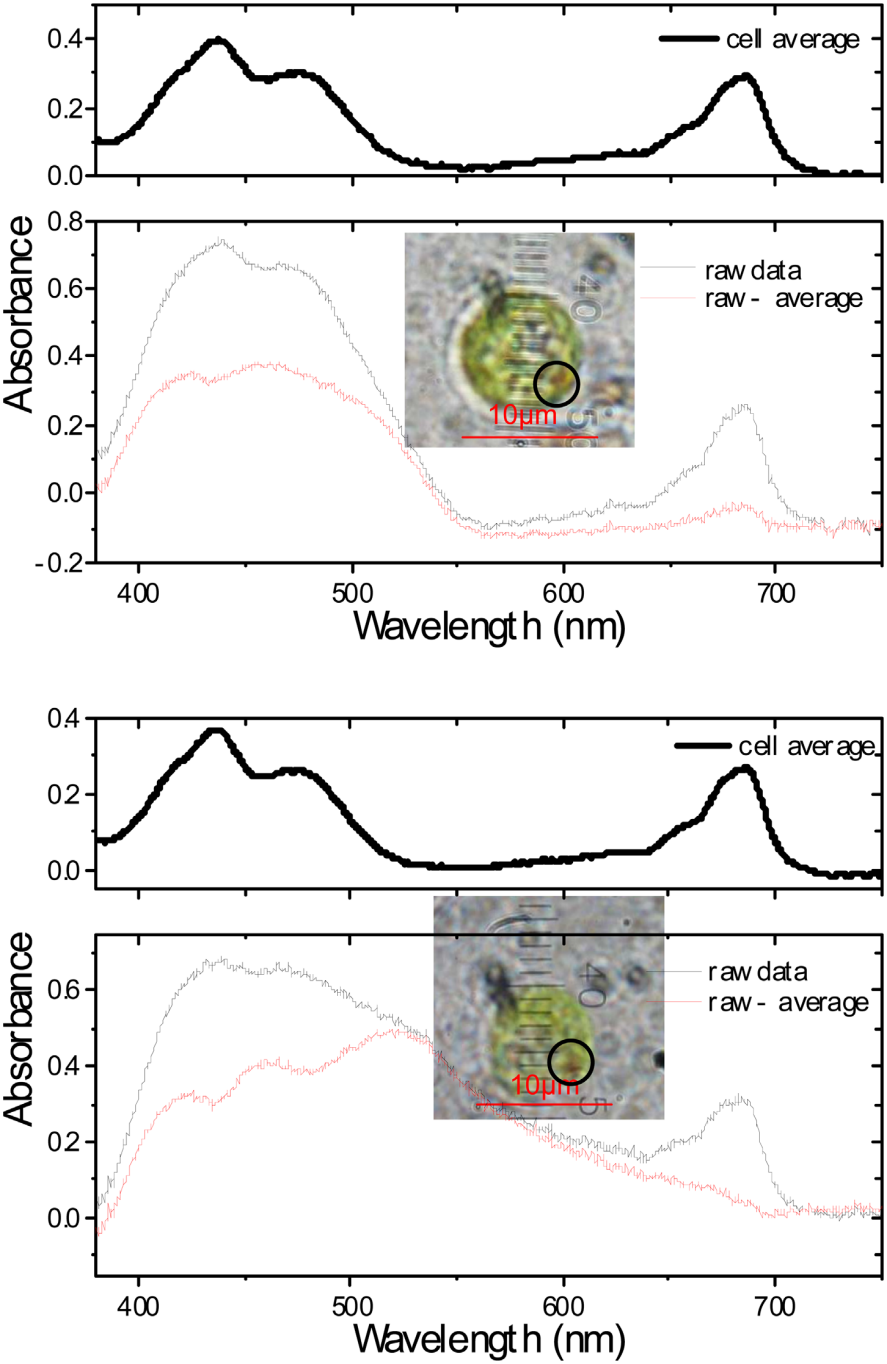
Absorbance spectra of 0.16 *μ*m × 0.6 *μ*m area (division unit) on the eyespot within two single live cells obtained by the 2D-1D conversion method. The estimated absorbance spectra (red lines) of the eyespot are calculated by subtracting the average spectra (thick black lines) from the raw spectra (thin black lines). The average spectra within the cells are calculated by the method in Appendix 2[B]. Insets: The color photographs of the live cells, where the eyespot is located on the edge of the cell within the circle.

Fig 3 shows absorbance spectra of the eyespot within single live cells shown in the insets in Fig 3, located in the region enclosed with the circle. The eyespot is a light-sensitive organelle as small as 1 *μ*m. We intentionally selected such a cell where the eyespot is located at the edge of the cell on the focal plane that the contamination of the spectrum by dominant absorption of chlorophyll is minimized. Even on these optimum conditions suitable for measuring the eyespot absorbance, background subtraction is necessary to extract estimated spectra of the eyespot. Here, we subtracted the average absorbace spectrum of each cell from the raw spectra at and around the eyespot and picked up such a spectrum that has the lowest 680-nm Chl *a* Q-band peak. The estimated absorbance spectra are similar to the previous observations of the eyespot apparatus isolated and purified from *Chlamydomonas* *reinhardtii*, either to Ref. [18] or to Ref. [19]. It is noted that the eyespot spectra are redshifted significantly (as large as 20 nm or more) compared with those for pigments extracted from eyespots of *Chlamydomonas* *reinhardtii* [19, 20] probably due to interaction with protein surroundings.

**Fig 4:**
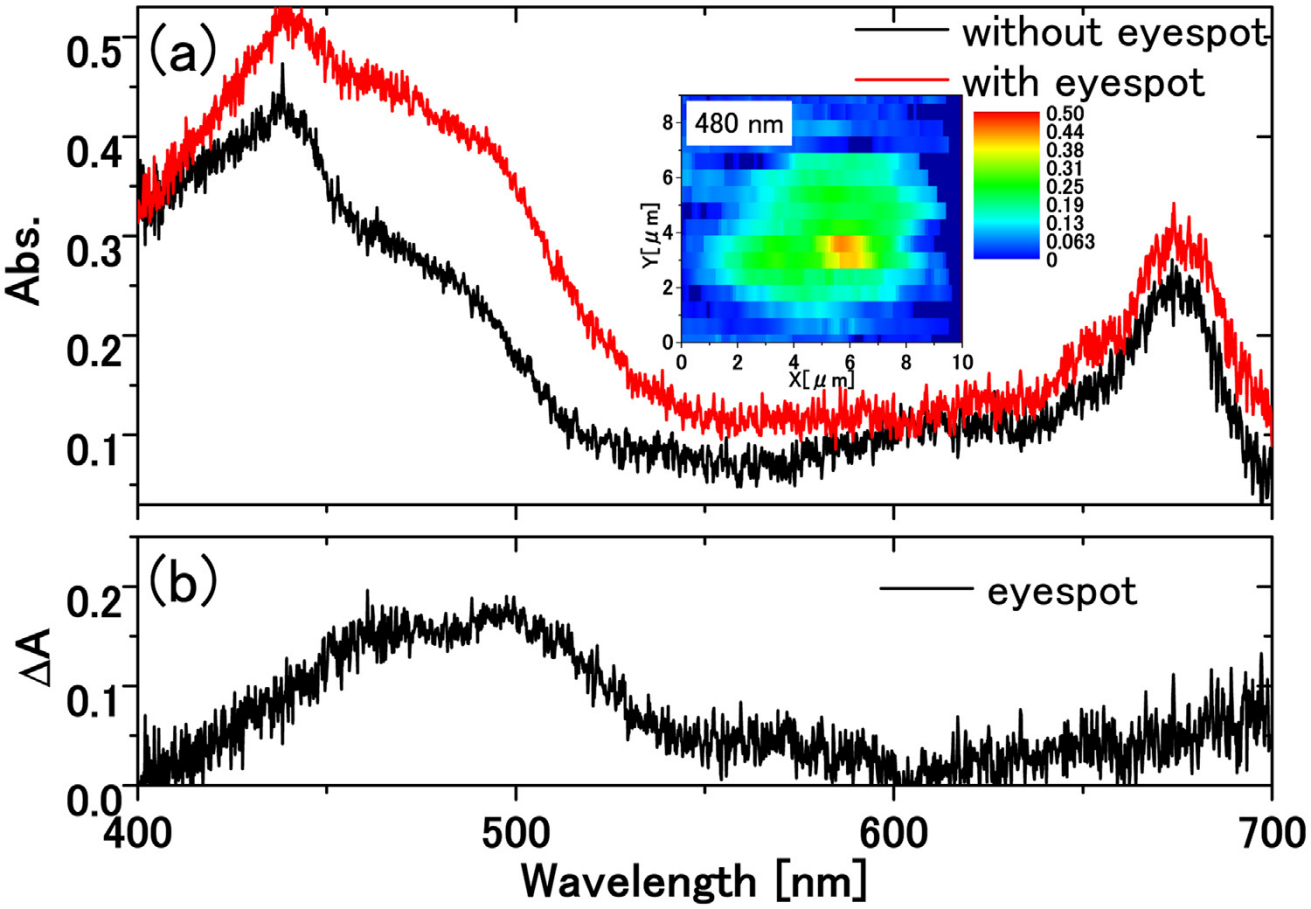
Absorbance spectrum of the eyespot within a live cell (II). Absorbance spectra of 0.10 *μ*m × 0.6 *μ*m area (division unit) within a single live cell obtained by the 2D-1D conversion method. (a) Absorbance spectra with and without an eyespot taken at the position over the red-yellow color region and over the adjacent green region, respectively, in the absorbance image of a single live cell at *λ* = 480 nm displayed in the inset. (b) Absorbance spectrum of the eyespot obtained as the difference spectrum between with and without the eyespot.

By this method, we can obtain the eyespot absorbance spectra even without selection of specifically oriented cells. Fig 4 shows absorbance spectrum of the eyespot within a single live cell where the eyespot is centered on the cell on the focal plane. The inset in Fig 4(a) clearly highlight the position of the eyespot in the absorption spectral image *A*(*x*, *y*, *λ* = 480 nm). In Fig 4(b) we obtained the eyespot absorbance as the difference spectrum between the spectra with and without the eyespot. The acquisition of the absorbance spectrum of the eyespot *in vivo*, i.e., within a normal live cell has been rare as long as we know. Usual approach has been to use a chlorophyll-defficient mutant to avoid contamination of chlorophyll absorption [18].

**Fig 5:**
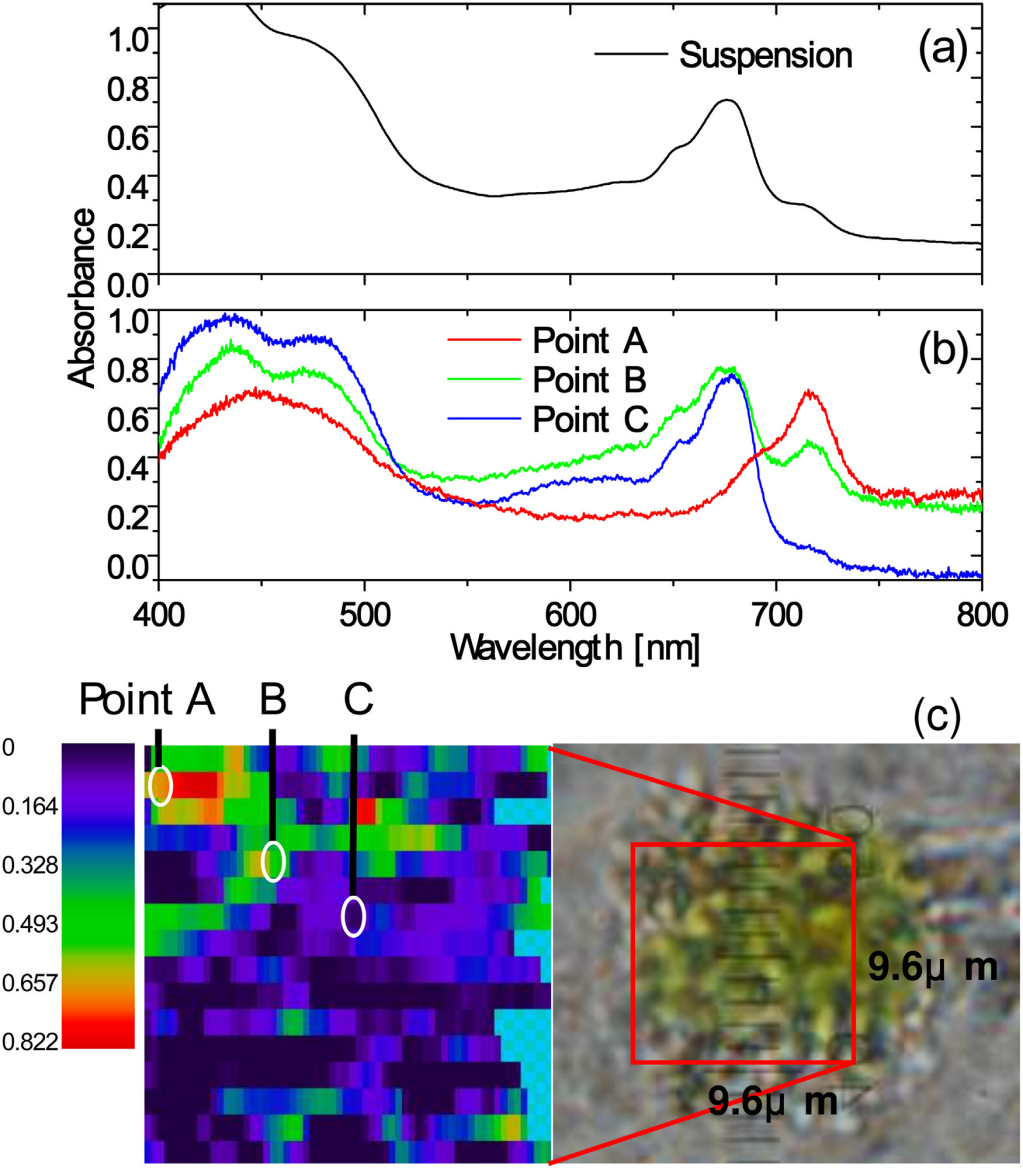
The 715-nm absorption peak localized within a single cell, measured by the 2D-1D conversion method. (a) Absorption spectrum of a cell suspension after under hydrogen generation conditions. (b) Position-dependent variation of absorbance spectra of 0.16 *μ*m × 0.6 *μ*m area (division unit) within a single cell obtained by the 2D-1D conversion method. (c) Right: The image of the cell for single-cell absorbance measurement in (b) picked up from the cell suspension with a 715 nm absorption peak. Left: A(x,y,*λ* = 715 nm) for the area enclosed by the red square within the cell. The local spectra at the positions A, B, and C are displayed in (b).

*C*. *reinhardtii* is attracting because of its hydrogen photoproduction property on the anaerobic condition. The most frequently used method to obtain the anaerobic condition is to use a sulfur-deprived *C*. *reinhardtii* culture [16]. In this culture, oxygenic photosynthesis is gradually deactivated under photoirradiation because reconstruction of photosynthetic proteins from photodamage is prevented. As a result, respiratory consumption of oxygen exceeds photosynthetic production of oxygen for the anaerobic condition to be realized to initiate hydrogen photoproduction. In this experiment, we found that after hydrogen generation the absorption spectra of the cell suspension change occasionally such that a new absorption peak appears around 715 nm on the longer wavelength side of Chl *a* (680 nm), as shown in Fig 5(a). This characteristic change in the absorption spectra do not always occur even on the same experimental conditions. Only known is that an anaerobic condition is necessary for occurrence of a 715-nm peak. The frequency is about one out of three cell suspensions. In addition, increase in the irradiation light intensity tends to enhance the probability to give rise to the 715-nm peak. Because of lacking for fully quantitative measurements so far, the necessary and sufficient condition has not yet been identified. Similar peaks were previously observed for *Euglena* after aging in the dark [21] or *Ginkgo biloba* [22], but the formation mechanism of the peak is not yet understood.

Only from this data, it is difficult to judge whether this change is due to the cell or to the culture solution. It is also unknown whether new kinds of pigments with single/double absorption peak are synthesized or protein structures surrounding pigments are modified. If the former is the case, there is several possibilities as follows. A well known photoreceptor pigment in higher plants, phytochrome, which regulates circadian rhythms, is known to have absorption in this wavelength region but is known to be absent from green algae [23]. Therefore this might be an unknown photoreceptor or such a pigment as redshifted Chl *f* [24] or structurally modified Chl *a* [25].

Thus, we applied absorption spectral imaging to single cells in this suspension. Fig 5(b) shows the result for one of the cells in the suspension. The absorption spectra changed from one site to another within the cell such that a single, isolated 715-nm peak between 600 nm and 800 nm was observed at certain positions within the cell. Along with the fact that the 715-nm peak disappeared when pigments were extracted from the cells with ethanol, the 715-nm peak is not caused by synthesis of a new kind of pigments (known from ethanol treatment) or by splitting of Chl *a* Q-band, but it is most likely to be caused by redshift of Chl *a* Q-band due to interaction with modified protein surroundings. This conclusion is consistent with the hypothesis in Ref. [21], and is difficult to be drawn without spatially resolving single cell absorbance.

## How to calculate Average Single Cell Absorbance from Cell Suspension Absorbance

**Fig 6:**
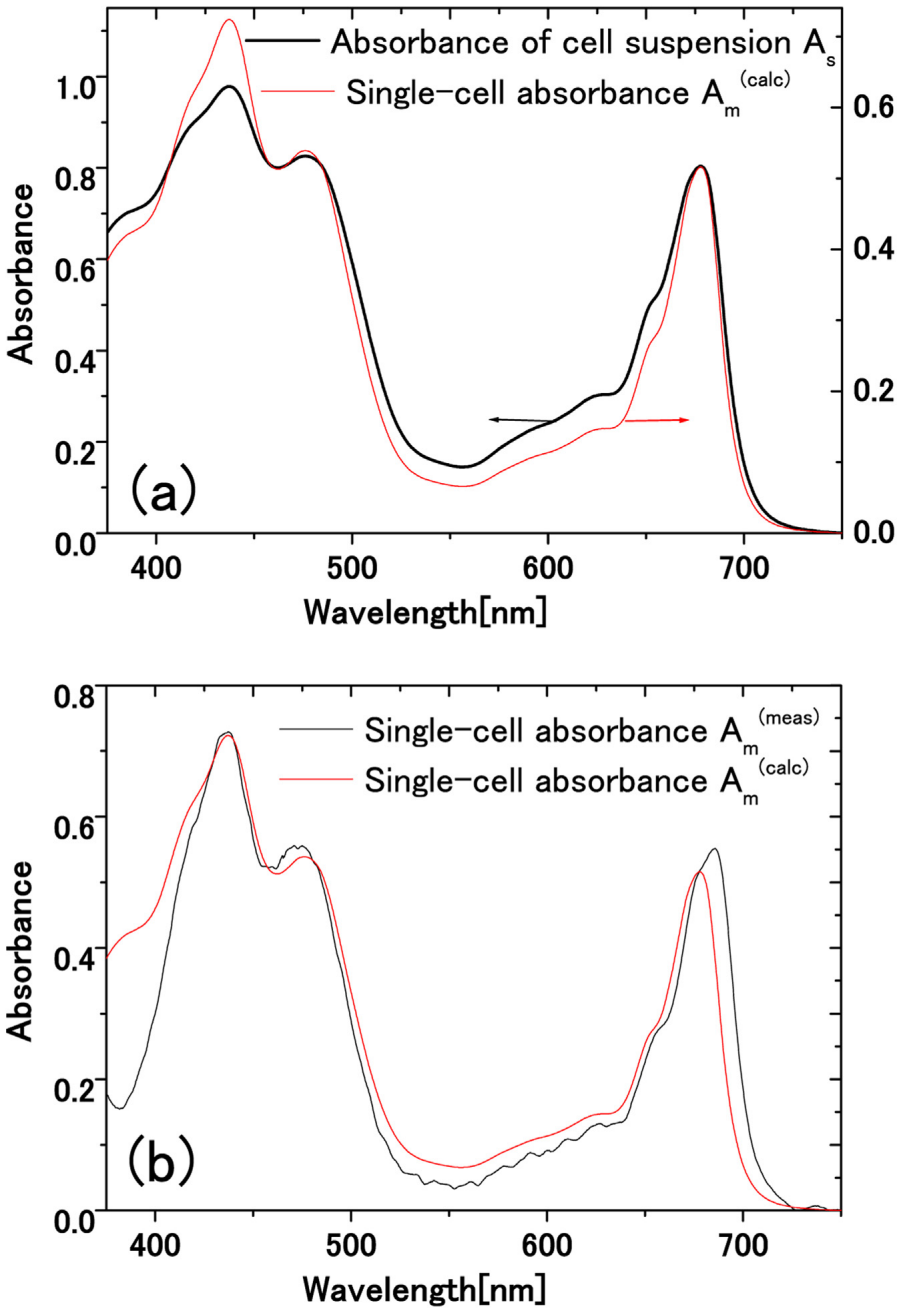
Comparison between single cell absorbance calculated from cell suspension absorbance and that averaged over 100 single-cell measurements. (a) Black: absorbance of a cell suspension of 5-mm path length measured with an integrating sphere. Red: maximum local absorbance calculated from the absorbance of the 5-mm cell suspension. (b) The maximum local absorbance of a single living cell, (black line) averaged over 100 single live cells measurement assuming a chloroplast of average diameter 7.42 *μ*m, and (red line) calculated from the absorbance of the cell suspension.

It is remarkable that the peak local absorbance in a single living cell is sometimes close to unity as shown in Fig 5(b), well comparable with that of cell suspensions as shown in Fig 6(a) (black line). We measured the spatially resolved absorbance *A*(*x*, *y*, *λ*) of 100 single live cells from the suspension to obtain the maximum local absorbance of the single cell, averaged over 100 single live cells in Fig 6(b) (black line) [Appendix 2 and Supporting Information (S1 Text, S1–S3 Figs)].

From the results in Fig 6, the average number of Chl *a* per cell can be evaluated by two ways using the molecular extinction coefficient of Chl *a*, 89.8 (mM)^−1^cm^−1^ [26, 27] as follows: We obtained 1.06 fmol/cell from the cell suspension absorbance using the cell number density *n*_c_ = 1.67 × 10^10^ cell/L, the path length *L* = 5 mm, and *Abs* = 0.797, while 1.46 fmol/cell was obtained from the single cell maximum absorbance *Abs* = 0.502 using the chloroplast diameter *d* = 7.42 *μ*m [Appendix 3]. This discrepancy indicates that *evaluation from cell suspensions usually leads to underestimation for the average number of pigments per cell or the average number density of pigments in the suspension*. In short, the cell concentration in cell suspensions is usually adjusted to get the absorbance close to unity at 680 nm. Since individual cells have maximum absorbance not far from unity, the cell concentration is inevitably so low as to allow a significant part of light to be transmitted through the suspension volume without blocked by any cells. This transmitted light reduces the absorbance. This fact is relevant to the well known package effect, but a fully quantitative analysis as given below has been difficult because of an insufficient knowledge about single-cell absorbance.

**Fig 7:**
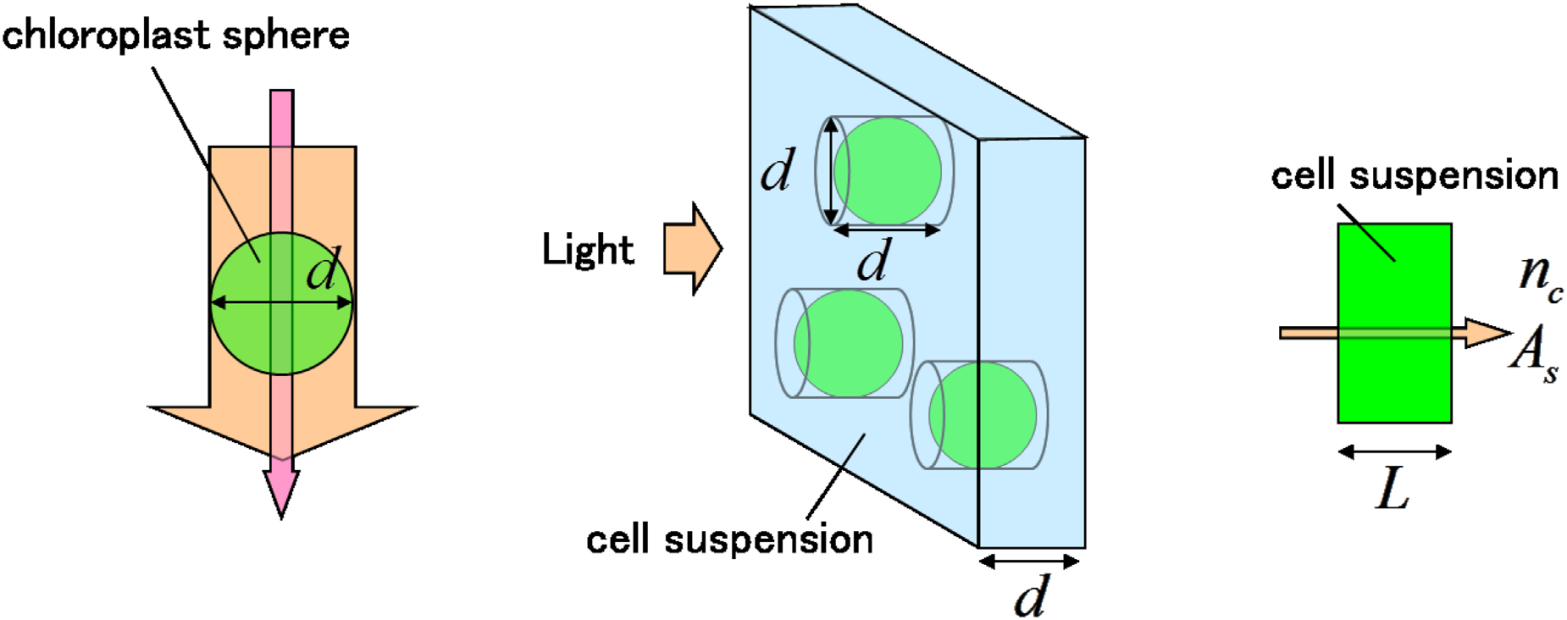
Model for relating the single cell absorbance to the cell suspension absorbance. *T*_sphere_:transmittance of the chloroplast sphere as shown in the left. *α*:absorption coefficient within the chloroplast. *d*:diameter of the chloroplast sphere. *n*_*c*_: cell number density in the cell suspension. *x*:chloroplast coverage ratio in the layer of the thickness *d* *T*_*d*_:transmittance of the layer of thickness *d* in the cell suspension *A*_s_:absorbance of the cell suspension *L*:thickness of the cell suspension *A*_m_:maximum local absorbance of a single cell for the light transmitted through the center of the cell

Let us calculate the absorbance of the 5-mm cell suspension on the basis of the absorbance of individual cells. For this purpose, we assume the following simplest model as depicted in Fig 7:

1. All the cells are a perfect sphere with the same diameter.
2. The light incident on the cell is neither refracted nor scattered (The refractive index of the cell is the same as that of the culture solution).
3. All the cell has a single spherical chloroplast. Light absorption is caused solely by a chloroplast within the cell. Thus, the cell sphere is regarded as the chloroplast sphere.
4. Pigments are uniformly and isotropically distributed within the chloroplast (homogeneous absorbance).
5. The cells are uniformly dispersed in the solution.

Since *Chlamydomonas* has a single chloroplast in each cell, we can equivalently consider the absorbance of the chloroplast suspension. For both macro- and micro-experiments, contribution of scattering to the absorbance spectra is neglected because small-angle forward scattering is known to be dominated in microalgae suspensions [10] and 100% and 48% out of the whole forward scattering light loss is collected, respectively, with the integrating sphere in the macro-measurement and with the NA0.85 objective lens in the micro-measurement. Here, the refractive index of the sphere is assumed to be very close to unity as usual [7], since microalgal cells are known to have a refractive index about 1.05 [10].

In fact, there is a distribution in the cell size against the assumption 1) and *Chlamydomonas reinhardtii* is known to have a cup-shaped chloroplast [15] for each cell against 3) and 4). How to deal with these problems is described in Supporting Information (S1 Text).

Transmittance of a homogeneously pigmented sphere (chloroplast) of diameter *d* and absorption coefficient *α* against a light ray having the same cross section as the sphere (i.e., when a cylindrical shape of ray which exactly hits the sphere such that the sphere is inscribed in a light ray cylinder) is calculated as

$$\begin{array}{ccc} T_{\text{sphere}} & = & \frac{\int_{0}^{d/2}e^{-2\alpha \sqrt{{\left(d/2\right)}^{2}-r^{2}}}rdr\int_{0}^{2\pi }d\theta }{\pi {\left(d/2\right)}^{2}}\\ & = & \frac{2}{\beta }\left(\frac{1}{\beta }-\frac{e^{-\beta }}{\beta }-e^{-\beta }\right) \end{array}$$ (1)

with *β* = *αd*. A cell suspension of thickness *L* is regarded as a stack of thin layers of thickness *d*. Transmittance of each layer is given by

$$\begin{array}{ccc} T_{d} & = & 1-x+xT_{\text{sphere}}, \end{array}$$ (2)

where *x* is the chloroplast cross sectional coverage ratio at each layer given by

$$\begin{array}{ccc} x & = & \pi {\left(d/2\right)}^{2}n_{c}d \end{array}$$ (3)

with *n*_c_ as the number density of cells in the suspension. Then, the absorbance of the suspension is calculated from

$$\begin{array}{ccc} A_{s} & = & -\log_{10}T_{d}^{L/d}. \end{array}$$ (4)

Since *β* = *αd* is obtained from the maximum local absorbance *A*_m_ = *β*/ln10 of a single cell, one can calculate the absorbance *A*_s_ of the cell suspension.

If this is inversely solved, one can obtain $A_{\text{m}}^{\left(\text{calc}\right)}$ from *A*_s_ as follows. From Eqs (2) and (4),

$$\begin{array}{ccc} T_{\text{sphere}} & = & (10^{-A_{s}d/L}+x-1)/x \end{array}$$ (5)

Eq (1) is expressed as

$$\begin{array}{ccc} \left(T_{\text{sphere}}/2\right)\beta^{2}+\left(\beta +1\right)e^{-\beta }-1 & = & 0. \end{array}$$ (6)

Given the value for *T*_sphere_, Eq (6) is numerically solved to obtain *β*. This inverse procedure gives the average (maximum local) absorbance of single cells. The average absorbance thus obtained is a good measure to be compared with each individual cell absorbance to judge if it has characteristic features deviating from the average.

From the absorbance of the *L* = 5 mm cell suspension in Fig 6(a) (black line) with *n*_c_ = (1.67 ± 0.04) × 10^4^/mm^3^ and *d*_ave_ = 7.42 ± 1.53 *μ*m, the average maximum local absorbance of the single cell was calculated as in Fig 6(a) (red line). It is noticed that the absorbance of the suspension is slightly flattened compared with that of the single cell, demonstrating the package effect. This calculated single-cell maximum local absorbance is displayed also in Fig 6(b) (red line) to be compared with that averaged over 100 single live cells using the process in Appendix 2 [2] and Supporting Information (S1 Text, S1–S3 Figs), shown in Fig 6(b) (black line). Agreement is reasonable, so that the formula relating the single cell absorbance and the cell suspension absorbance is confirmed to be valid.

## Conclusions

The absorbance spectral imaging microscopy system was developed and applied to single live algal cells, *C*. *reinhardtii*. For rapid acquisition of *A*(*x*, *y*, *λ*), the 2D-1D conversion fiber assembly was introduced to successfully detect the local absorbance spectra of a 1-*μ*m organelle, eyespot, under a major background of chlorophyll absorption within a live cell. In addition, the 715-nm absorbance peak in the cell suspension which occasionally appeared after hydrogen photogeneration was investigated for each single cell in the suspension to find the single 715-nm absorption peak was locally distributed within the cell.

By comparing the absorbance between the cell suspension and the single cell, it is experimentally confirmed that evaluation of the average number of chlorophylls per cell from cell suspensions usually results in underestimation due to the package effect. For accurate estimation from suspensions, the formula to calculate the average single cell absorbance from the cell suspension absorbance was presented and executed. The result showed reasonable agreement with the single cell absorption measurement. The Eq (4) with Eqs (1), (2) and (3) relates *A*_s_(absorbance of a cell suspension), *L*(thickness of the suspension), *α*(average absorption coefficient of the cell), *n*_c_(cell number density in the suspension), and *d*(average cell diameter = average chloroplast diameter). This is useful, for example, to obtain a good estimate for the cell number density from other experimental parameters, since it is difficult to obtain the accurate cell number density by counting cells without use of automated counting devices such as a Coulter counter.

Presently three- or four-times scan is needed so that the measurement time for acquiring *A*(*x*, *y*, *λ*) of a single cell amounts to several tens of seconds. This can be readily improved to a few seconds if a CCD and an imaging spectrograph with the vertical size larger than 16 mm are employed. By increasing the power of the light incident on the cell, it is within our scope to reduce the exposure time to less than 1 second to enable dynamical absorption spectral imaging of unicellular organisms under flagellar movement without fixing them.

## Appendices

### Appendix 1

**Fig 8:**
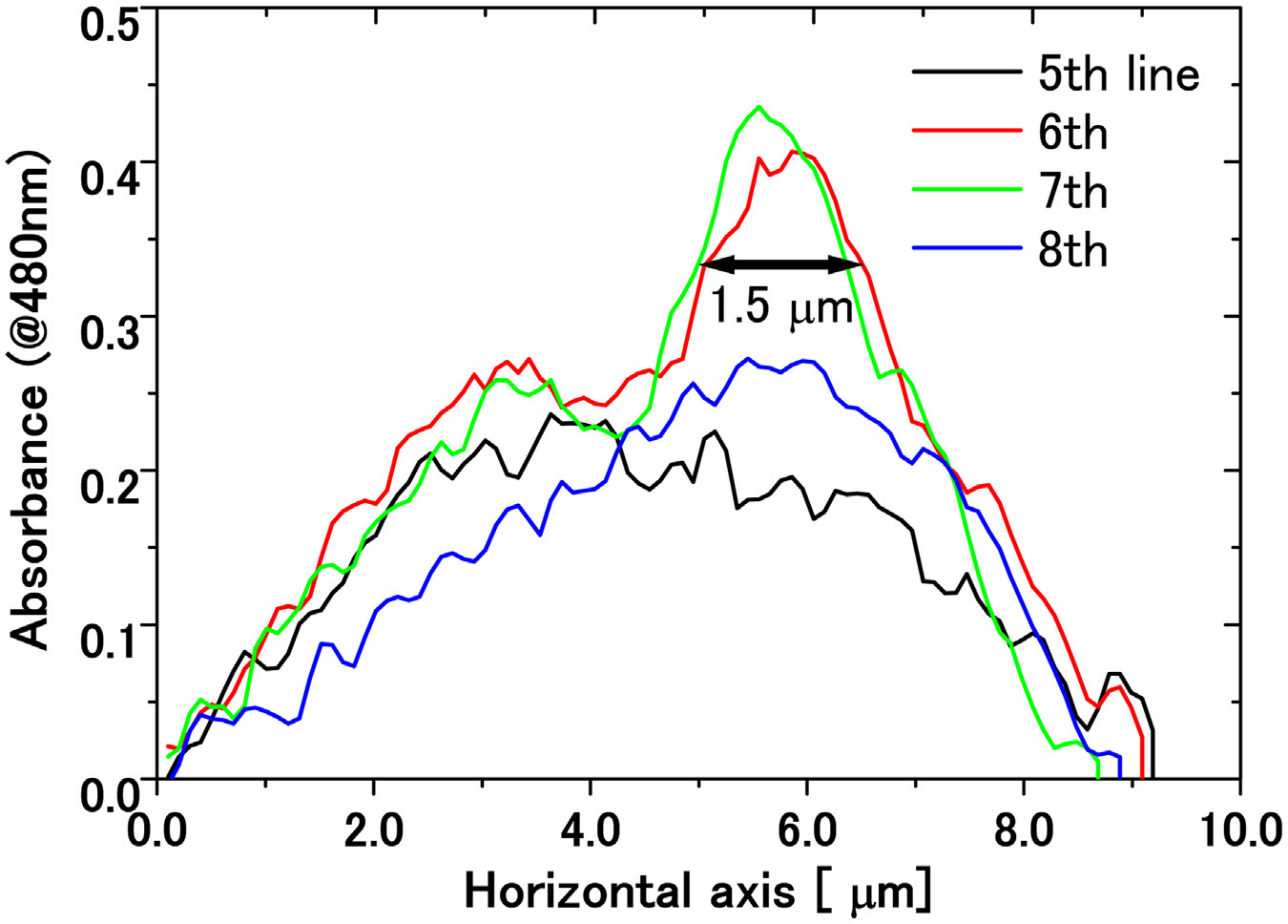
Experimental data to estimate the spatial resolution of the system. Absorbance at 480 nm around the eyespot in the inset in Fig 4 as a function of position. The FWHM of the absorbance image of the eyespot is about 1.5 *μ*m.

The spatial resolution of the absorbance image is estimated as follows. The spatial distribution of the absorbance image of the eyespot in Fig 4 is displayed in Fig 8.

The FWHM (full width at half maximum) of the absorbance profile for the eyespot is estimated to be 1.5 *μ*m. If we assume that the eyespot has a spherical shape of 1 *μ*m diameter, its absorbance *A*(*x*) as a function of the distance *x* from its center is expressed by $A(x)\propto 2\sqrt{a^{2}-x^{2}}$, where *a* is the radius of the sphere with *a* = 0.5 *μ*m. Then, the FWHM of *A*(*x*) is $\sqrt{3}a=0.866\;\mu \text{m}$. Further we assume that the eyespot absorbance profile is expressed by the Gaussian function with the same FWHM. The point spread function is also assumed to be the Gaussian with the FWHM of *b*. Here, we define the resolution by *b*, which is determined from the equation $\sqrt{0.866^{2}+b^{2}}=1.5$ to be *b* = 1.22 *μ*m.

### Appendix 2

The average absorbance *A*_sphere_ of the whole single cell is obtained by two ways, leading to slightly different values, as follows:

1. Irradiating light on the whole cross section of the cell as shown in Fig 7,
  $$\begin{array}{ccc} A_{\text{sphere}}^{\left(1\right)} & = & -\log_{10}T_{\text{sphere}}=-\log_{10}\frac{2}{\beta }\left(\frac{1}{\beta }-\frac{e^{-\beta }}{\beta }-e^{-\beta }\right). \end{array}$$ (7)
  The maximum local absorbance *A*_m_ is evaluated as $A_{\text{m}}^{\left(\text{meas}\right)}=\beta /\text{ln}\;10$.
2. Averaging the local absorbance $A_{\text{loc}}=2\alpha \sqrt{{\left(d/2\right)}^{2}-r^{2}}/\text{ln}\;10$ within the cell to get
  $$\begin{array}{ccc} A_{\text{sphere}}^{\left(2\right)} & = & \frac{\;\int \int \;A_{\text{loc}}rdrd\theta }{\;\int \int \;rdrd\theta }=\frac{2\beta }{3\ln10}. \end{array}$$ (8)
  The maximum local absorbance *A*_m_ is evaluated as $A_{\text{m}}^{\left(\text{meas}\right)}=\beta /\text{ln}\;10=3A_{\text{sphere}}^{\left(2\right)}/2$.

### Appendix 3

The average number of Chl *a* per cell, *n*_m_, is evaluated from the cell suspension absorbance as

$$\begin{array}{ccc} n_{m}\left[\text{mol}/\text{cell}\right] & = & \frac{Abs}{\epsilon \left[L/(\text{mol}\cdot \text{cm})\right]\times L\left[\text{cm}\right]\times n_{c}\left[\text{cell}/L\right]}\\ & = & 1.06\times 10^{-15}\left[\text{mol}/\text{cell}\right] \end{array}$$

where *Abs* is the absorbance of the cell suspension (*Abs* = 0.797), *ϵ* is the molecular extinction coefficient of Chl *a* (*ϵ* = 89.8×10^3^ M^−1^cm^−1^) [26, 27], *L* is the path length (*L* = 0.5 cm), and *n*_c_ is the cell number density (*n*_c_ = 1.67 × 10^10^ cell/L).

It is evaluated from the single cell maximum absorbance as

$$\begin{array}{ccc} n_{m}\left[\text{mol}/\text{cell}\right] & = & \frac{Abs}{\epsilon [L/(\text{mol}\cdot \text{cm})]\times d[\text{cm}]}\times (\frac{4}{3}\pi {\left(d/2\right)}^{3}\times 10^{-3})\;\left[L/\text{cell}\right]\\ & = & 1.46\times 10^{-15}\left[\text{mol}/\text{cell}\right] \end{array}$$

where *Abs* is the maximum absorbance of the single cell (*Abs* = 0.502) and *d* is the chloroplast diameter (*d* = 7.42 ×10^−4^cm).

## Supporting Information

S1 Text Detailed experimental and analysis methods in Sec. IV. (DOC)

S1 Fig a: Absorbance of the cell suspension in a 5-mm cell before and after single-cell absorbance. b: Absorbance of the cell suspension in a 5-mm cell averaged before and after single-cell absorbance measurement.
(DOC)

S2 Fig a: Images where the number of cells was counted. b: Distribution of the number of cells per image. c: Images where the diameters of cells were evaluated. d: Distribution of the cell diameter in the suspension. e: Distribution of the cell volume in the suspension. f: Absorbance of the cell suspension and the single-cell absorbance calculated from it, the same as in Fig 6(a) in the text. Here, the absorbance of the cell suspension in S1b Fig is shifted by 0.0494 to show zero absorbance at 750 nm. Similarly, *A*_m_, which was calculated from *A*_s_ without shift, is shifted by 0.0208.
(DOC)

S3 Fig a: Microscope images of individual cells with their absorbance spectral images *A*(*x*, *y*, *λ* = 437 nm). b: Distribution of *α* for 100 cells. c: Single-cell absorbance averaged for 100 cells. The spectrum is shifted by -0.0116 to show zero absorbance at 750 nm. d: Comparison between single-cell absorbance calculated from cell suspension absorbance and that averaged over 100 single-cell measurements, the same as in Fig 6(b).
(DOC)

S4 Fig a: 0th order CCD image of the 1D fiber array on the slit. b: 1st order diffraction (spectrally dispersed) image of light from the 1D fiber array. c: Absorbance spectral image of the cell having 715 nm peak in Fig 3(c). d: The area enclosed by the red circle is magnified to show a rectangle unit constituting the image.
(DOC)
