# Supplementary material for: Scan-Free Absorbance Spectral Imaging A(x, y, λ) of Single Live Algal Cells for Quantifying Absorbance of Cell Suspensions
Source: PLoS One. 2015 Jun 10;10(6):e0128002. doi: 10.1371/journal.pone.0128002 (PMC4465668; doi:10.1371/journal.pone.0128002)
Supplement: S1 Text — (DOC) [file pone.0128002.s001.doc]

Supporting Information S1

This file describes details of the experimental and analysis methods in Sec. IV:

“IV. How to calculate average single-cell absorbance from cell-suspension absorbance.”

To be concrete, “How to obtain three spectra in Figs. 6(a) and 6(b)” and how to measure four relevant physical quantities,

*A*_s_：absorbance of cell suspension

*n*_c_ [cells/mm^3^]：cell number density in the suspension

*d* [μm]: average diameter of cells in the suspension

α[μm ^-1^ ] : average absorption coefficient within a single cell

are described. Experimentally measued values for *n*_c_ , *d*, and　α are statistically distributed. How to handle such a distribution is also described.

Contents

1. The absorbance *A*_s_ of the cell suspension in Fig. 6(a).

2. The maximum local absorbance *A*_m_^(cal)^ of the single cell calculated from the *A*_s_ in Figs. 6(a) and 6(b).

2-1. How to measure the cell number density *n*_c_ in the cell suspension.

2-2. How to measure the average cell diameter *d*_s_ in the cell suspension.

2-3. Calculation of *A*_m_^(calc)^ using *A*_s_, *n*_c_, and *d*_s_ in Fig.6(a).

3. The maximum local absorbance *A*_m_^(meas)^ of the single cell, obtained from the absorbances of individual single cells *A*(*x*,*y*,λ), in Fig. 6(b).

3-1. Absorbance spectral imaging *A*(*x*,*y*,λ) of individual cells.

3-2. How to measure the average cell diameter *d*_a_ of the individual cells whose *A*(*x*,*y*,λ) ’s were measured.

3-3. The average α and *A*_m_^(meas)^ of the single cell.

------------------------------------------------------

4. Minimum area of the local absorbance determined by the CCD pixel size and the core diameter of the 2D-1D fiber.

The maximum local absorbance *A*_m_ is defined by *A*_m_=β/ln10=αd/ln10, which means absorbance when light is transmitted through the maximum pass (=diameter) in the assumed spherical shape of chloroplast.

We assumed the following simplest model in the text:

1) All the cells are a perfect sphere with the same diameter.

2) The light incident on the cell is neither refracted nor scattered (The refractive index of the cell is the same as that of the culture solution).

3) All the cell has a single spherical chloroplast. Light absorption is caused solely by a chloroplast within the cell. Thus, the cell sphere is regarded as the chloroplast sphere.

4) Pigments are uniformly and isotropically distributed within the chloroplast (homogeneous absorbance).

5) The cells are uniformly dispersed in the solution.

In fact, however, there is a distribution in the cell size against 1) and *Chlamydomonas reinhardtii* is known to have a cup-shaped chloroplast for each cell against 3) and 4). (Therefore, depending on observation direction, it looks spherical with fully packed or cup-shaped with a transparent region.)

This SI describes how to reconcile a cell-size distribution and a non-spherical shape of chloroplast with the assumptions 1), 3) and 4).

**Experimental procedure:**

Before absorbance measurement of individual cells.

[1] Absorbance measurement of the 5-mm cell suspension by 5 times using an integrating sphere

[2] The cell number density measurement from 341 images on pra”parats sampled from the suspension.

[3] The cell diameter measurement from 191 images on pra”parats sampled from the suspension.

[4] Absorbance measurement of 100 individual single cells from the cell suspension.

Since the single-cell absorbance measurement took about a half day, the condition of the cell suspension might change during the measurement. In order to avoid the effect of such a change in the suspension, characteristic physical quantities of the suspension were measured before and after the single-cell absorbance measurement and averaged throughout.

After absorbance measurement of individual cells.

[5] Absorbance measurement of the 5-mm cell suspension by 5 times using an integrating sphere

[6] The cell number density measurement from 258 images

[7] The cell diameter measurement from 230 images.
