# Supplementary material for: Scan-Free Absorbance Spectral Imaging A(x, y, λ) of Single Live Algal Cells for Quantifying Absorbance of Cell Suspensions
Source: PLoS One. 2015 Jun 10;10(6):e0128002. doi: 10.1371/journal.pone.0128002 (PMC4465668; doi:10.1371/journal.pone.0128002)
Supplement: S1 Fig — a: Absorbance of the cell suspension in a 5-mm cell before and after single-cell absorbance. b: Absorbance of the cell suspension in a 5-mm cell averaged before and after single-cell absorbance measurement. (DOC) [file pone.0128002.s002.doc]

**1. The absorbance *A*_s_ of the cell suspension in Fig. 6(a).**

**Measurement:**

1. A 20mL of cell suspension in a screw cap glass vial was stirred, and an aliquot (about 1.5 mL) from the suspension was injected into a 5-mm path-length cell to be measured with spectrophotometer using an integrating sphere.

2. The suspension in the 5-mm cell was returned to the vial.

3. Repeating 1 and 2 by 5 times.

**Analysis:**

The absorbance of the cell suspension was measured by 5 times before and after the single cell absorbance measurement and then 10 data were averaged.

**Result:**

Fig.S1a

Absorbance of the cell suspension in a 5-mm cell before and after single-cell absorbance.

Fig.S1b

Absorbance of the cell suspension in a 5-mm cell averaged before and after single-cell absorbance measurement.

Using *n*_c_=1.67×10^4^ cells/mm^3^, the suspension in the 5-mm cell is estimated to contain 2.51×10^7^ cells.
