# Supplementary material for: Scan-Free Absorbance Spectral Imaging A(x, y, λ) of Single Live Algal Cells for Quantifying Absorbance of Cell Suspensions
Source: PLoS One. 2015 Jun 10;10(6):e0128002. doi: 10.1371/journal.pone.0128002 (PMC4465668; doi:10.1371/journal.pone.0128002)
Supplement: S2 Fig — a: Images where the number of cells was counted. b: Distribution of the number of cells per image. c: Images where the diameters of cells were evaluated. d: Distribution of the cell diameter in the suspension. e: Distribution of the cell volume in the suspension. f: Absorbance of the cell suspension and the single-cell absorbance calculated from it, the same as in Fig 6(a) in the text. Here, the absorbance of the cell suspension in S1b Fig is shifted by 0.0494 to show zero absorbance at 750 nm. Similarly, A m, which was calculated from A s without shift, is shifted by 0.0208. (DOC) [file pone.0128002.s003.doc]

**2. The maximum local absorbance *A*_m_^(calc)^ of the single cell calculated from the *A*_s_, in Figs. 6(a) and 6(b).**

**2-1. The cell number density *n*_c_ measurement in the cell suspension**

**Experiment:**

1. The cell suspension, whose absorbance was measured with the integrating sphere, was well stirred.

2. A 10 µL was taken from the suspension and dropped on a slideglass with a micropipette. It was covered with a cover slip of 18 mm×18 mm as a pra”parat for observation with a microscope using a ×10 objective.

3. Randomly sampled images of 1.09 mm×0.81 mm area on the pra”parat were captured, and the number of cells per area was counted by the original program developed by Y. N..

4. This process was repeated for 5 pra”parats to obtain 341 images before the single-cell abs. experiment. Similarly, 258 images for 5 pra”parats after the experiment.

**Analysis:**

Total 599 images were analyzed to reach the total count of 211663 cells to obtain 353.36 cells as the average number per image. 16.3% of the total area of the samples under the cover slips was counted and the total volume of the suspension where the cell number was counted was calculated using the measured thickness of the suspension, 24um, between the cover slip and the slideglass.

**Result:**

As a result, we obtained a distribution of the number of cells per image with average of 353.36 cells/image and standard deviation of 185.91 cells/images as shown in the graph. The total number of cells per all 599 images is 353.36×599=211663 cells with the standard deviation 185.91×√599=4550.05 cells (from the central limit theorem), which is converted into *n*_c_=16738.5 cells/mm^3^ with the standard deviation 359.8 cells/mm^3^ in the total sampled volume of the cell suspension.

**Example:**

The following images of 1.09 mm×0.81 mm area are typical examples in the analysis. Cells marked by magenta by the program were counted. The graph shows a distribution of the number of cells per image with average of 353.36 cells/image and standard deviation of 185.91 cells/images. The standard deviation was by 10 times larger than that expected for a Poisson statistics shown in the blue curve.


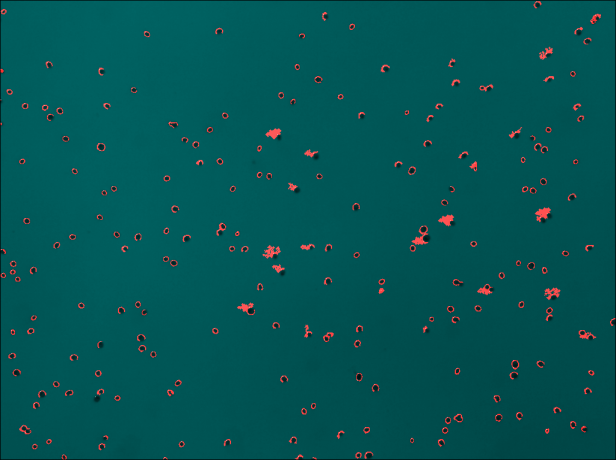

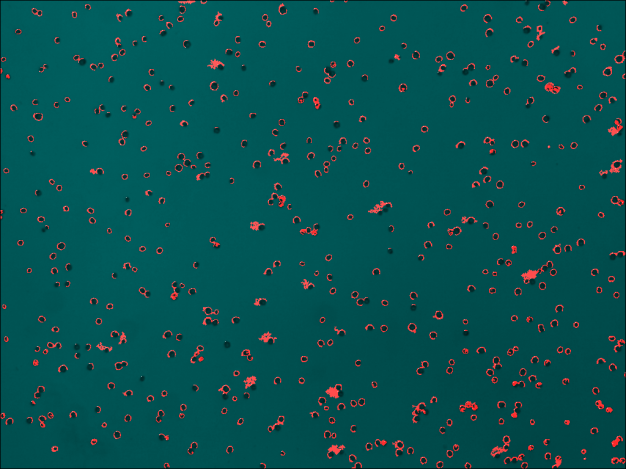

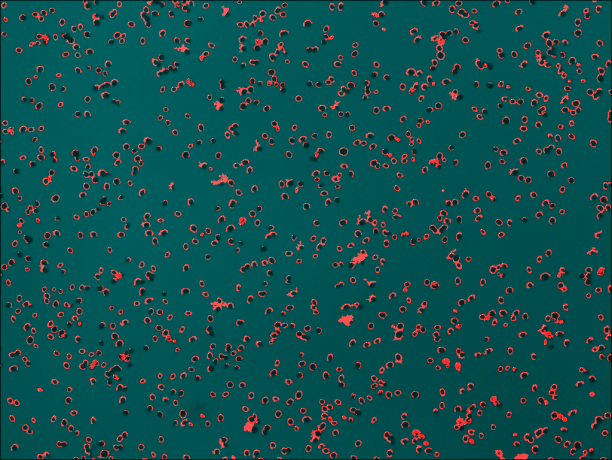


222 cells / image

553 cells / image

1104 cells / image

Fig.S2a

Images where the number of cells was counted.

Fig.S2b

Distribution of the number of cells per image.

**2-2. The average cell diameter *d*_s_ measurement in the cell suspension**

**Experiment:**

1. A small amount from the suspension used for absorbance measurement was taken and diluted to the concentration suitable for the cell diameter measurement within the field of view observed with ×100 objective.

2. A 10 uL from the diluted suspension and almost the same amount of agar were dropped on a slide glass, and then covered with a cover slip as a pra”parat for observation with a microscope.

3. Appropriate images magnified by 100 times were taken for 5 to 7 pra”parats. 191 and 230 images were obtained before and after the single-cell abs. experiment, respectively.

**Analysis:**

The cell diameters *d*_s_ were evaluated from the image files using the original program developed by Y. N.

**Result:**

461 cells were measured to obtain the average cell diameter of 7.30 μm before the experiment and 385 cells were to obtain 7.04 μm after the experiment. The total average was *d*_s_=7.19 μm±1.30 μm.

**Example:**

The following is typical examples for analyzed images of 768 ×1024 pixels with 0.00013 mm/pixel 0.09984 ×0.13312 mm^2^.


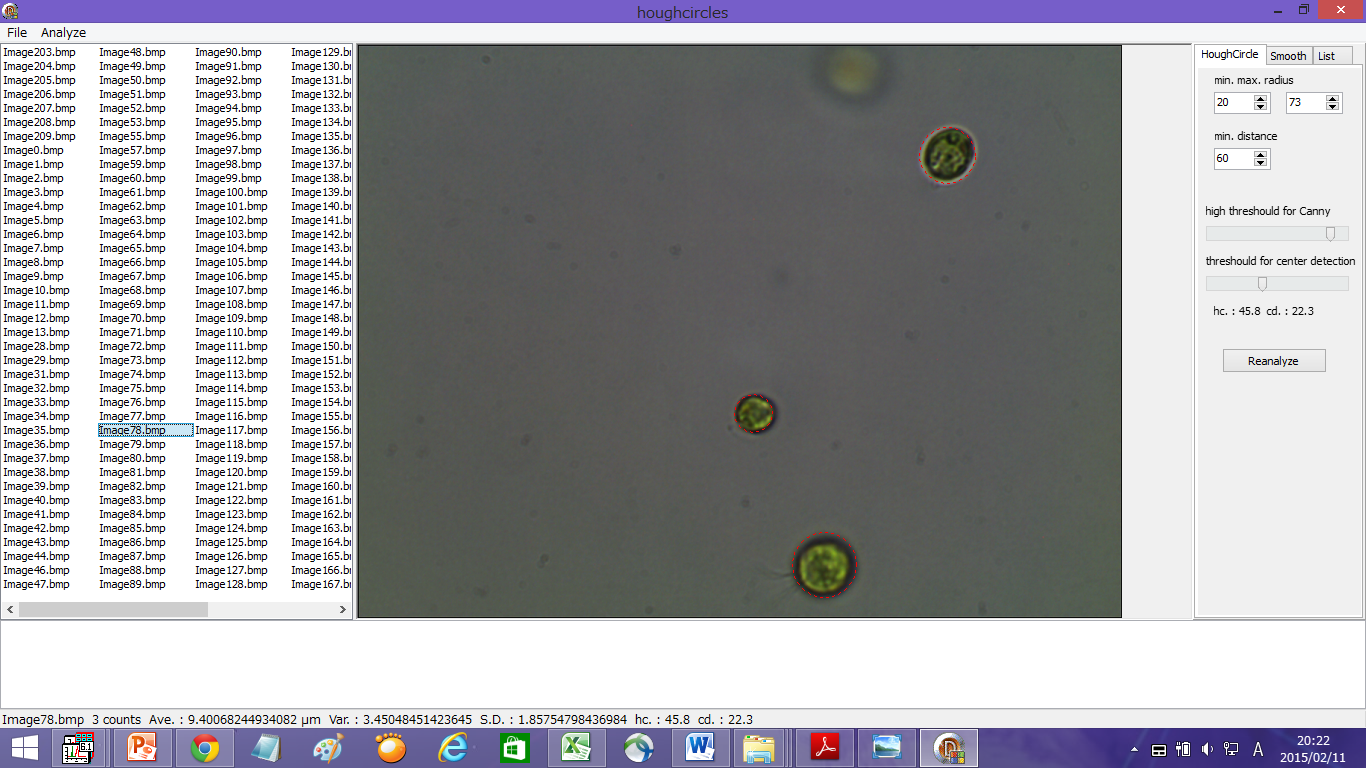

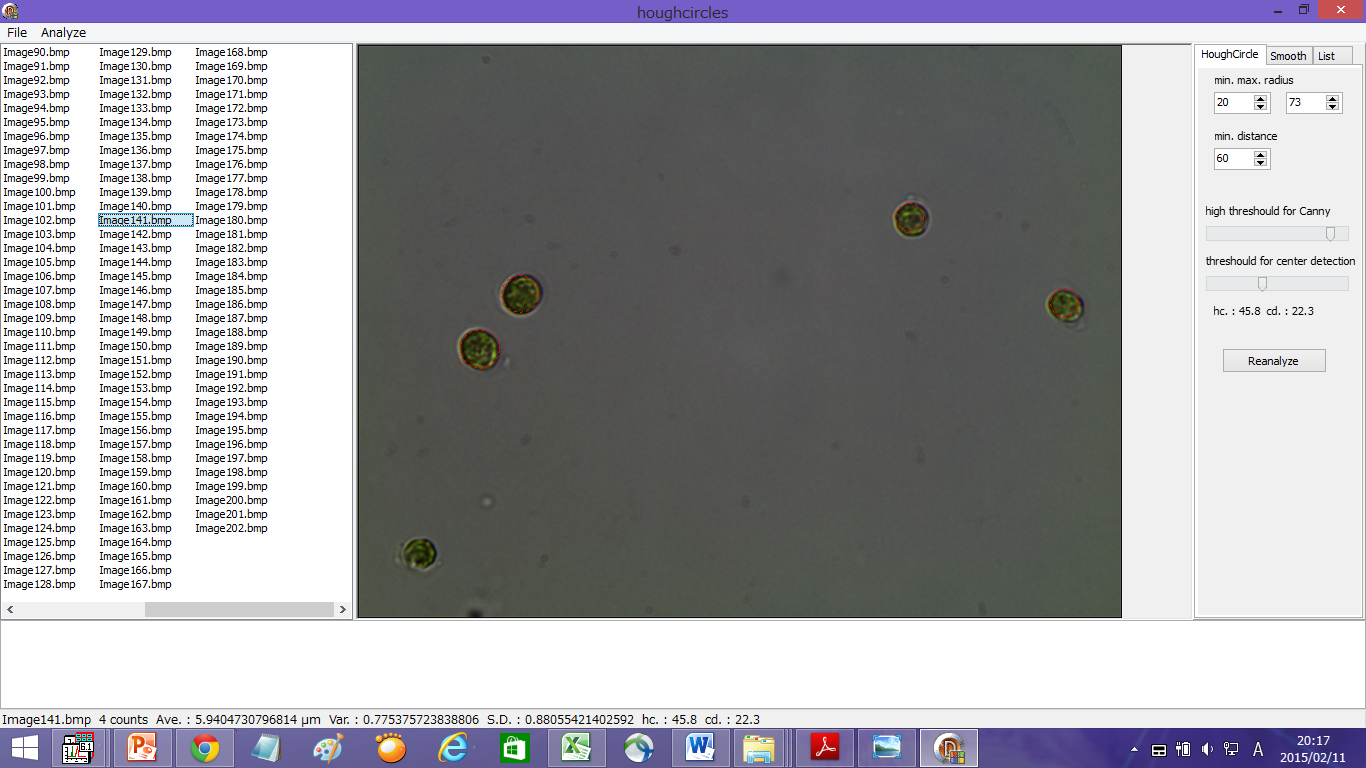


average diameter 5.94µm for 4 cells

average diameter 9.40µm for 3 cells


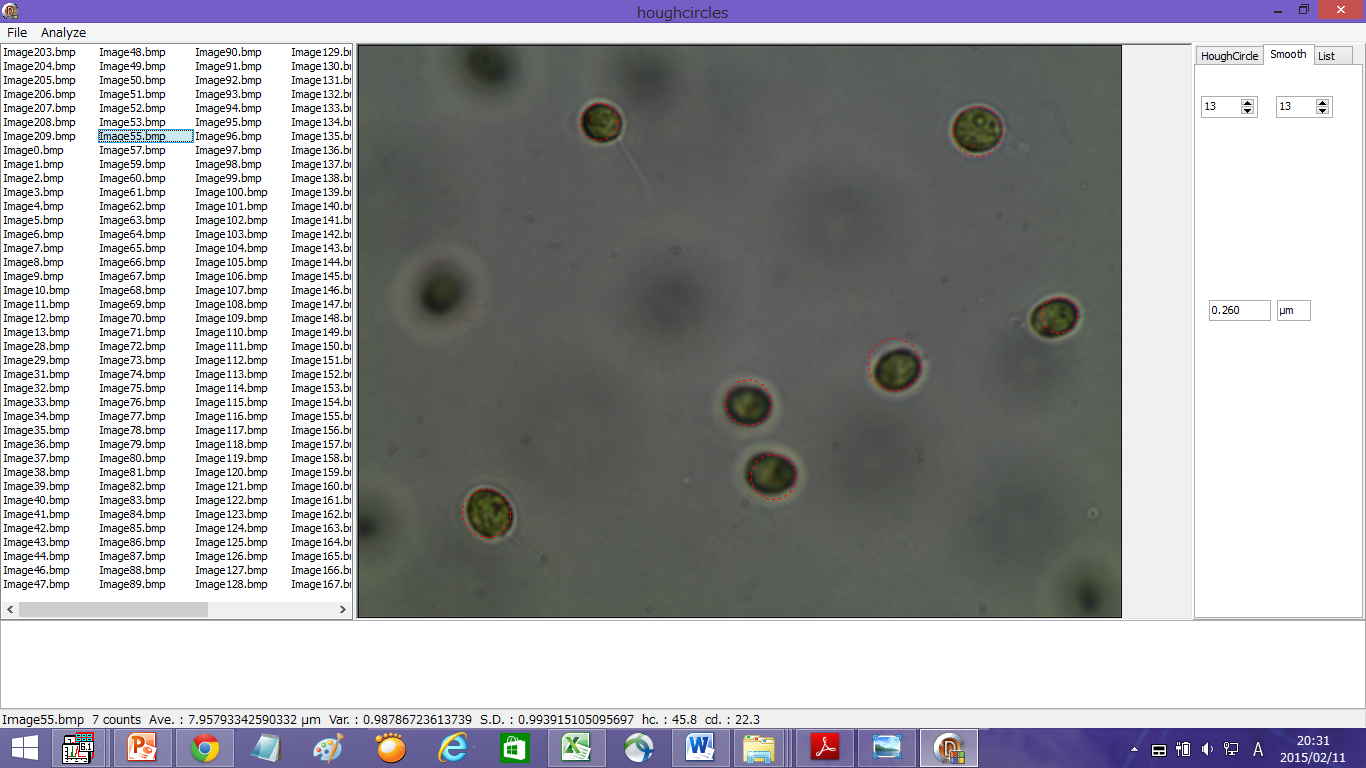


Fig.S2c

Images where the diameters of cells were evaluated.

average diameter 7.96µm for 7 cells

Fig.S2d

Distribution of the cell diameter in the suspension.

**Analysis:**

As shown in the graph, the cell-to-cell varience in the diameter was significant (The standard deviation of the diameter distribution is 1.30 um. ) so that use of the single, average diameter may cause an estimation error in calculating the single cell absorbance from the cell suspension absorbance. Although it is desirable to incorporate this distribution to the analysis, we adopted the following alternative method here in order to avoid complexity in the analysis.

The absorbance *A* of the cell suspension with a certain path length *L* is proportional to α*L*（*A*∝α*L*）with absorption coefficient α. Since α is proportional to the number density of cells, it is also proportional to the total volume of the cells (chloroplasts) . That is, as the first approximation, *A* is proportional to the total volume of the cells. Then, even if the cell diameter is distributed symmetrically with respect to the average diameter, the larger half of cells in the diameter distribution contributes more to the absorbance than the smaller half in the distribution. In order to take this effect into account, we converted the cell-diameter distribution to the cell-volume distribution to determine the average cell volume. Form this, the average effective diameter was deduced.

**Result (from volume distribution):**

Here is the cell (chloroplast) volume distribution. The estimated average effective diameter was *d*_s_=7.42 µm±1.53 µm. This value was used for calculation of both *A*_m_^(calc)^ and *A*_m_^(meas)^ from the experimental data.

Fig.S2e

Distribution of the cell volume in the suspension.

**2-3. Calculation of *A*_m_^(calc)^ using *A*_s_, *n*_c_, and *d* in Fig.6(a).**

Using Eqs. (1) to (4) with the inverse procedure (5) and (6) in the text, the single-cell absorbance was calculated in Fig. 6(a) as shown below.

Fig.S2f

Absorbance of the cell suspension and the single-cell absorbance calculated from it, the same as in Fig.6(a) in the text. Here, the absorbance of the cell suspension in Fig.S1b is shifted by 0.0494 to show zero absorbance at 750 nm. Similarly, *A*_m_, which was calculated from *A*_s_ without shift, is shifted by 0.0208.
