# Supplementary material for: Scan-Free Absorbance Spectral Imaging A(x, y, λ) of Single Live Algal Cells for Quantifying Absorbance of Cell Suspensions
Source: PLoS One. 2015 Jun 10;10(6):e0128002. doi: 10.1371/journal.pone.0128002 (PMC4465668; doi:10.1371/journal.pone.0128002)
Supplement: S3 Fig — a: Microscope images of individual cells with their absorbance spectral images A(x, y, λ = 437 nm). b: Distribution of α for 100 cells. c: Single-cell absorbance averaged for 100 cells. The spectrum is shifted by -0.0116 to show zero absorbance at 750 nm. d: Comparison between single-cell absorbance calculated from cell suspension absorbance and that averaged over 100 single-cell measurements, the same as in Fig 6(b). (DOC) [file pone.0128002.s004.doc]

**3. Absorbance of single cells:**

**The maximum local absorbance *A*_m_^(meas)^ of the single cell, obtained from the absorbances of individual single cells *A*(*x*,*y*,λ), in Fig. 6(b).**

**3-1. Absorbance spectral imaging *A* (*x*,*y*,λ) of individual cells.**

**Experiment:**

1. Three pra”parats A, B, C were prepared in a similar way to the cell-diameter measurement 2-2.

2. With a ×100 objective, a baseline transmission spectrum *T*_0_(*x*,*y*,λ) without cells was measured per 10 cells and transmission spectra *T*(*x*,*y*,λ) of cells as close to the baseline position as possible were measured.

3. About 33 measuring cells were picked up from each (sample) pra”parat to measure total 100 cells. The space-resolved absorbance spectra *A*(*x*,*y*,λ) were calculated from *T*(*x*,*y*,λ) and *T*_0_(*x*,*y*,λ). Photos of cells whose absorbance were measured were taken with a digital camera through the eyepiece lens of the microscope. .

**Example:**

*A*(*x*,*y*,λ=437 nm)’s and microscope images of three cells:


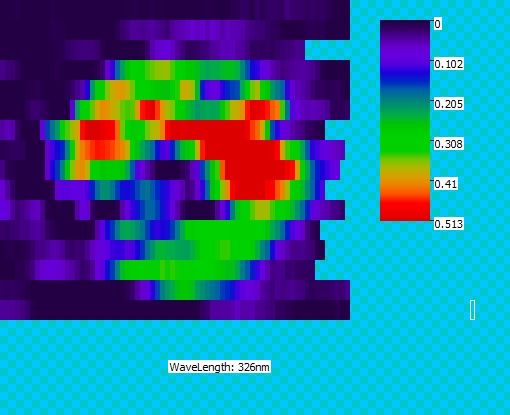

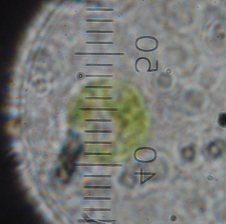


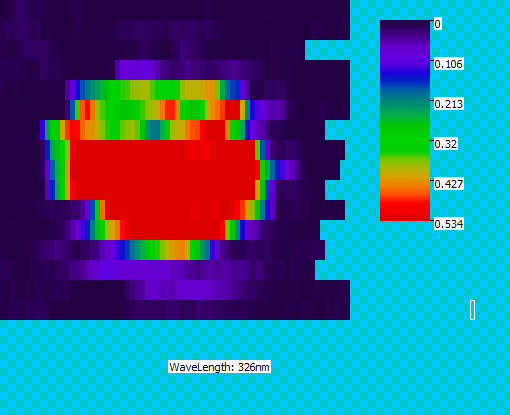

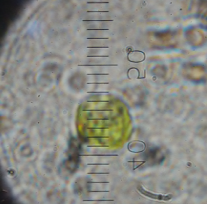


Fig.S3a

Microscope images of individual cells with their absorbance spectral images A(x,y,λ=437 nm).


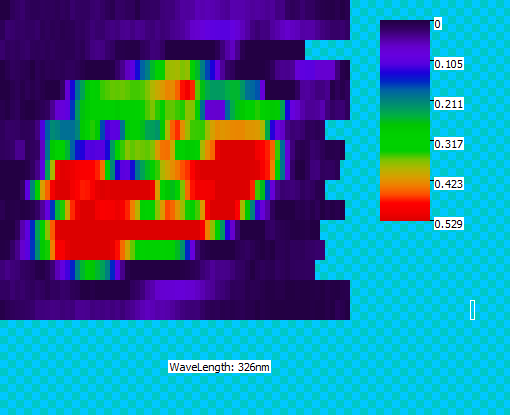

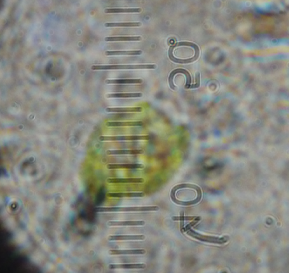


**3-2．The average cell-diameter *d*_a_ of the individual cells in the single absorbance measurement.**

**Analysis:**

The photos of the 100 cells magnified by ×100 taken in 3-1 were analyzed with the open software ImageJ to obtain the cell diameter. If the shape of a cell is an ellipse, the diameter *d* was calculated as *d*=√major axis×√minor axis.

**Result:**

As a result, the average diameter was *d*_a_=7.057±1.407 μm. Similarly to the diameter analysis for the cell suspension, the diameter distribution was converted to the volume distribution to obtain the average effective diameter, *d*_a_=7.327±1.506 μm. (The average *d*_a_ was not directly used in the text. The distribution of *d* in 100 cells shows a good measure to judge whether a typical group of cells are sampled from the suspension. )

**3-3. The average absorption coefficient α and the maximum local absorbance *A*_m_^(meas)^ in Fig. 6(b) of the single cell, from measurement of single-cell absorbance.**

**Analysis:**

1. The average absorbance *A*_sphere_ of the cell was evaluated from the local absorbance of the cell with the method in Appendix 2[2], where the maximum local absorbance *A*_m_ of the cell is given by 3 *A*_sphere_ /2. The shape of the chloroplast was assumed to be spherical or elliptical, so that the whole local absorbances were summed up within the individual chloroplast diameter *d* estimated in 3-2 even if a transparent region was included there. In other words, a minimum circle or ellipse, which encloses any spot where absorbance at 437 nm is finite (chlorophyll a B-band), was taken as the shape.

2. As a result, the absorption coefficient $\alpha_{i}$ of the $i$th cell with $d_{i}$ was estimated from $A_{m(i)}$ by . Then, the average α of the cell was estimated by $\alpha=\frac{\sum_{i} \alpha_{i}d_{i}^{3}}{\sum_{i} d_{i}^{3}}$. The maximum local absorbance *A*_m_^(meas)^ of the single cell was finally obtained by *A*_m_^(meas)^=α*d*_s_/ln10.

**Result:**

α(437 nm)=0.185±0.051 µm was obtained with the distribution shown in the graph below.

Fig.S3b

Distribution of α for 100 cells.

Fig.S3c

Single-cell absorbance averaged for 100 cells. The spectrum is shifted by -0.0116 to show zero absorbance at 750 nm.

Fig.S3d

Comparison between single-cell absorbance calculated from cell suspension absorbance and that averaged over 100 single-cell measurements, the same as in Fig. 6(b).
