# Supplementary material for: Scan-Free Absorbance Spectral Imaging A(x, y, λ) of Single Live Algal Cells for Quantifying Absorbance of Cell Suspensions
Source: PLoS One. 2015 Jun 10;10(6):e0128002. doi: 10.1371/journal.pone.0128002 (PMC4465668; doi:10.1371/journal.pone.0128002)
Supplement: S4 Fig — a: 0th order CCD image of the 1D fiber array on the slit. b: 1st order diffraction (spectrally dispersed) image of light from the 1D fiber array. c: Absorbance spectral image of the cell having 715 nm peak in Fig 3(c). d: The area enclosed by the red circle is magnified to show a rectangle unit constituting the image. (DOC) [file pone.0128002.s005.doc]

**4. Minimum area of the local absorbance determined by the CCD pixel size and the core diameter of the 2D-1D fiber.**

The minimum area of the local absorbance is given by 0.16µm×0.6µm or 0.1µm×0.6µm in Figs. 2,3,4, and 5 with ×100 objective.

This is because 50µm core with 60µm pitch of the 1D fiber is incommensurate with 16µm×16µm pixel size of the CCD camera. The minimum resolution for the X-axis of the CCD is determined by the 60µm diameter of the 1D fiber on the entrance slit of the spectrometer (the slit is fully open so that the slit width is equivalent to 60µm) since the horizontal axis of the CCD is for wavelength resolution. On the other hand, along the Y-axis of the CCD , 3.75 pixels (=60µm /16µm) are occupied by 1 fiber diameter (60µm) of the 1D fiber. As a result, the minimum area (division unit) is 0.16µm×0.6µm with ×100 objective.

0.1µm×0.6µm in Figs. 2 and 4 is due to difference in f=35cm (MS3504i) and f=10cm (SL100M) imaging spectrometers because the f=35cm spectrometer magnifies the slit image by 1.5 times on the output focal plane.

Actual resolution is so determined as to be given in Appendix 1, which is due to the limitation of NA in the optical system in addition to the effect above.

X


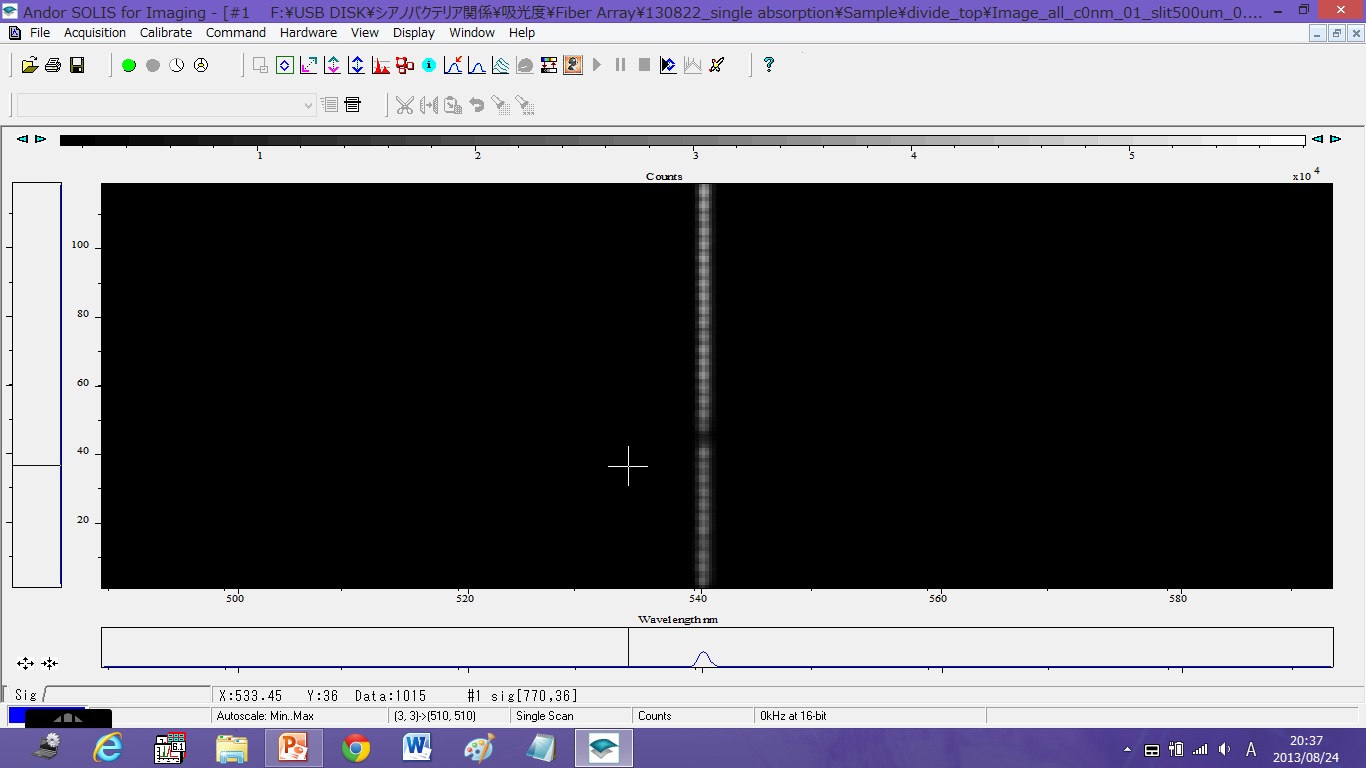


Y


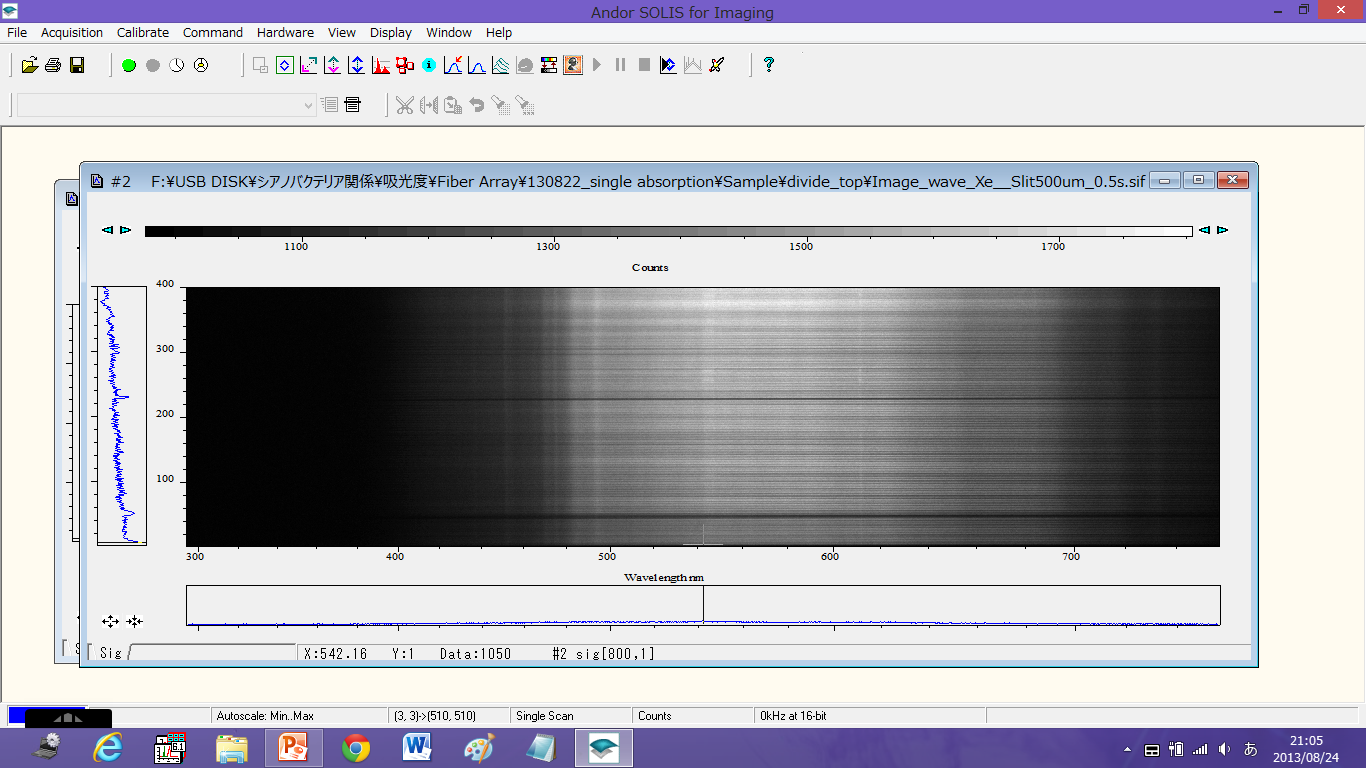


Fig.S4b

1^st^ order diffraction (spectrally dispersed) image of light from the 1D fiber array.

Y


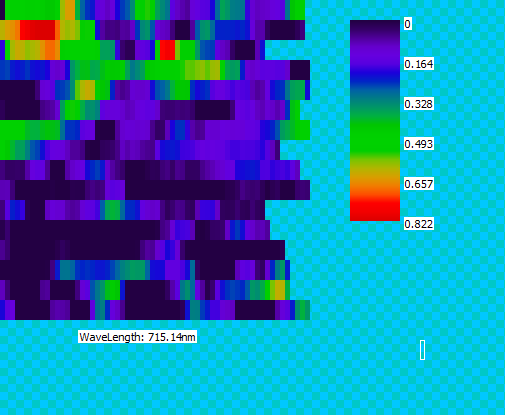

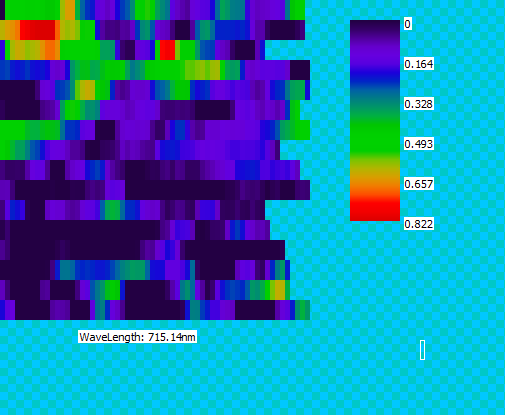


0.15µm

0.6µm

Fig.S4a

0^th^ order CCD image of the 1D fiber array on the slit.

Fig.S4c

Absorbance spectral image of the cell having 715nm peak in Fig.3(c).

Fig.S4d

The area enclosed by the red circle is magnified to show a rectangle unit constituting the image.
